# Pathways and molecules for overcoming immunotolerance in metastatic gastrointestinal tumors

**DOI:** 10.3389/fimmu.2024.1359914

**Published:** 2024-04-05

**Authors:** Qixin Gan, Yue Li, Yuejun Li, Haifen Liu, Daochuan Chen, Lanxiang Liu, Churan Peng

**Affiliations:** ^1^1Department of Radiology, First Affiliated Hospital of Hunan College of TCM (Hunan Province Directly Affiliated TCM Hospital), Zhuzhou, Hunan, China; ^2^Department of Cardiovascular Medicine, First Affiliated Hospital of Guangxi Medical University, Nanning, Guangxi, China; ^3^Department of Oncology, First Affiliated Hospital of Hunan College of TCM (Hunan Province Directly Affiliated TCM Hospital), Zhuzhou, Hunan, China

**Keywords:** gastrointestinal cancer, immunotolerance, immune escape, metastasis, pathway, molecule

## Abstract

Worldwide, gastrointestinal (GI) cancer is recognized as one of the leading malignancies diagnosed in both genders, with mortality largely attributed to metastatic dissemination. It has been identified that in GI cancer, a variety of signaling pathways and key molecules are modified, leading to the emergence of an immunotolerance phenotype. Such modifications are pivotal in the malignancy’s evasion of immune detection. Thus, a thorough analysis of the pathways and molecules contributing to GI cancer’s immunotolerance is vital for advancing our comprehension and propelling the creation of efficacious pharmacological treatments. In response to this necessity, our review illuminates a selection of groundbreaking cellular signaling pathways associated with immunotolerance in GI cancer, including the Phosphoinositide 3-kinases/Akt, Janus kinase/Signal Transducer and Activator of Transcription 3, Nuclear Factor kappa-light-chain-enhancer of activated B cells, Transforming Growth Factor-beta/Smad, Notch, Programmed Death-1/Programmed Death-Ligand 1, and Wingless and INT-1/beta-catenin-Interleukin 10. Additionally, we examine an array of pertinent molecules like Indoleamine-pyrrole 2,3-dioxygenase, Human Leukocyte Antigen G/E, Glycoprotein A Repetitions Predominant, Clever-1, Interferon regulatory factor 8/Osteopontin, T-cell immunoglobulin and mucin-domain containing-3, Carcinoembryonic antigen-related cell adhesion molecule 1, Cell division control protein 42 homolog, and caspases-1 and -12.

## 1 Introduction

Significant health concerns stem from GI system malignancies, which encompass diseases affecting the digestive tract and its supplementary organs, including colorectal, gastric, and hepatic cancers (1). These diseases are among the most common conditions diagnosed in both men and women. According to the 2020 Global Cancer Statistics, gastric cancer is ranked fourth, and colorectal cancer (CRC) is the second leading cause of cancer-related mortality globally (2). Furthermore, projections from CANCER TOMORROW indicate a significant rise in both new cases and deaths from GI cancers by 2040. Even with a slight decline in incidence rates due to progress in early detection techniques, mortality rates for patients with advanced, inoperable GI cancers remain alarmingly high. Amidst this scenario, the latest advancements in tumor immunotherapy have surfaced as a ray of hope for these advanced stages (3). Conventional treatments, such as surgery, chemotherapy, and radiation therapy, often lead to suboptimal outcomes, marked by local and distant recurrence. This inadequate response might be linked to the immune tolerance noted in certain patients. Therefore, investigating new treatments, notably immunotherapy, is considered a promising avenue for the effective control of these diseases.

A fundamental factor in these cancers’ progression is immune tolerance – a biological phenomenon where the immune system, in a paradoxical manner, neglects to identify and attack tumor cells. Consequently, cancer cells, by altering their antigen expression patterns, effectively impersonate the body’s cells, thereby utilizing the immune system’s tolerance mechanisms to their advantage. This adaptation is crucial for their survival and proliferation (4). In the cancer immunity cycle, where immune cells typically recognize and attack malignant cells, cancer cells hinder this process via immune evasion. This immune evasion is particularly impactful during the equilibrium phase of the cancer-immune interaction, where the immune system and cancer cells maintain a dynamic balance of power. The cancer cells’ aptitude to induce immune tolerance, similar to physiological tolerance observed in normal tissues, is a fundamental component of their survival strategy (5, 6). Thus, addressing these distinct immune evasion tactics is imperative for crafting potent therapeutic approaches against cancer. Such strategies hold the promise of revolutionizing cancer therapy, shifting the paradigm from conventional modalities like chemotherapy and radiation to more precise immunotherapies that leverage the immune system’s capabilities.

With this knowledge as a foundation, the intent of this review is to delve into the pathways and targets linked to immune tolerance in metastatic GI tumors. By dissecting these mechanisms, we strive to reveal potential groundbreaking therapeutic avenues, broadening the scope of treatment alternatives for these daunting cancers.

## 2 Immune microenvironment in GI cancers

The TME is a dynamic, intricate space comprising diverse immune cells—T and B lymphocytes, TAM, DC, NK cells, neutrophils, and MDSC. Additionally, stromal cells like CAF, pericytes, and mesenchymal stromal cells contribute to its complexity, fostering a microenvironment that actively shapes tumor behavior. Integral to the TME are elements such as the ECM, growth factors, cytokines, chemokines, and EV. The interaction with vascular networks, both blood and lymphatic, serves as a defining feature, orchestrating a complex interplay that influences the behavior of cancer cells and various cellular components within the microenvironment (7–9). Macrophages, often polarized to a pro-tumorigenic phenotype in the TME, contribute significantly to tumor progression and metastasis by supporting tumor cell survival, promoting angiogenesis, and suppressing antitumor immune responses. Conversely, CD8+ T cells, recognized for their potential to mediate tumor rejection, face substantial suppression in the TME due to a variety of immunosuppressive mechanisms, including checkpoint blockade and metabolic competition (10, 11). This nuanced exploration underscores the complexity of TME-tumor interactions and highlights the therapeutic potential of targeting these dynamics, particularly through strategies aimed at reactivating CD8+ T cell responses and modulating macrophage activity within the TME. Notable features like hypoxia, an acidic milieu (12), nerve-rich areas (13), mechanical factors (14), and an immune-suppressive environment collectively define the TME. These characteristics intricately contribute to the unique nature of the microenvironment, influencing tumor initiation, progression, and response to therapeutic interventions. Each constituent of the TME, from immune and stromal cells to environmental factors, holds significant functional roles. They actively participate in the initiation, development, invasive behavior, metastasis, and therapeutic response of tumors. The intricate interplay among these components forms a dynamic network that profoundly shapes the overall behavior and progression of cancer (15). Understanding the intricate details of the TME is paramount for developing targeted therapeutic strategies. By targeting specific elements within the TME, there exists a promising avenue for more effective cancer treatment. This is particularly crucial given the profound impact of the TME on tumor initiation, progression, and responses to various therapeutic modalities (16, 17).

In the intricate immune tapestry of gastric and CRCs, a spectrum of immune defenders orchestrates a multifaceted response. T cells, B cells, macrophages, natural killer cells, and dendritic cells collaborate, each with a distinct role (18). At the forefront of defense, cytotoxic T cells (CD8+) wield their prowess against intracellular threats, aiming to dismantle cancer cells in gastric and colorectal domains. Their precision and potency make them pivotal players in immune surveillance. Acting as conductors in the immune orchestra, T helper cells (CD4+) harmonize responses. From catalyzing antibody production to activating macrophages, their multifunctionality extends to orchestrating a cohesive defense, uniting immune forces against gastric and CRCs (19). Additionally, at the heart of adaptive immunity, B cells showcase versatility. They emerge as crucial antigen presenters, facilitating the orchestration of immune responses. As B cells mature into plasma cells, their dual role emerges — generating antibodies and secreting cytokines. This dynamic process amplifies the arsenal against cancer in gastric and colorectal contexts (20). Equally important, functioning as vigilant phagocytes, macrophages are instrumental in the immune theater. Recognizing and neutralizing intracellular threats, these cells stand resilient against cancer in gastric and colorectal environments. Their versatility extends to modulating the immune microcosm, shaping the battle against malignancies. As sentinels of both innate and adaptive immunity, natural killer cells assume a central role. Identifying and swiftly eliminating cancer cells in gastric and colorectal settings, their innate proficiency complements the orchestrated efforts of other immune actors. The balance they strike enhances the overall resilience against malignancies. Furthermore, in the intricate dance of immunity, dendritic cells take center stage as primary conductors. Serving as premier antigen-presenting maestros, they shape the immune symphony. Their regulatory prowess extends to both innate and adaptive realms, influencing immune evasion and molding the nuanced landscape of the tumor microenvironment in gastric cancer (21, 22). In the immune landscape of gastric and CRCs, diverse immune cell types collaborate, each with a unique role, constructing a complex and robust immune defense to combat the invasion of cancer cells.

The TME plays a pivotal dual role in the immune response. Initially, it suppresses tumor development, acting as a barrier against the early stages of tumor progression. However, as tumors advance, the TME undergoes a shift, transforming into a milieu that promotes a tumor-friendly environment. This dual action creates a paradoxical scenario, fostering immune tolerance and thereby facilitating further tumor advancement. The development of immune tolerance involves a complex interplay of factors. Tumors actively engage in competition for metabolites during their progression, leading to a heightened demand for resources. Additionally, they release extracellular vesicles and cytokines, contributing to the intricate orchestration of immune suppression. Furthermore, tumors strategically reduce antigen expression, impairing the recognition of cancerous cells by the immune system. Within the tumor microenvironment, the functionality, quantity, and distribution of cytotoxic immune cells are hindered, collectively culminating in the establishment of immune tolerance. Several key factors contribute significantly to the development of immune tolerance in the presence of tumors. The primary factor is the depletion of immune cells, which weakens the immune response against the growing malignancy (23). Functional inhibition of immune cells is equally crucial, as it impairs their ability to mount an effective antitumor response. Additionally, tumor-induced alterations within the TME play a significant role in shaping the immune landscape, creating an environment conducive to immune evasion (24). Collectively, these factors intertwine to establish a state of immune tolerance, allowing tumors to evade the immune system’s surveillance and thrive.

## 3 Pathways associated with immune tolerance in metastatic GI cancers

### 3.1 PI3K/AKT signaling pathway

Phosphatidylinositol 3-kinases (PI3Ks) are a diverse group of lipid kinases classified into three distinct classes—Class I, II, and III—each defined by unique structural and functional characteristics. The PI3K signaling pathway can undergo aberrant activation through several mechanisms: activating mutations or amplification of PI3K catalytic subunits, inactivation of the lipid phosphatase PTEN (phosphatase and tensin homolog), or amplification or mutation of cellular receptors. Notably, PTEN serves as a critical negative regulator within this pathway, countering the actions of PI3K/AKT signaling (25). Discovered in the 1970s, protein kinase AKT, also known as protein kinase B, was identified as an oncogene by the transforming retrovirus AKT-8, which was isolated from the AKR mouse thymoma cell line. The AKT gene occupies a central role in mediating cellular responses to various growth factors, cytokines, and other stimuli through its involvement in signaling cascades, thereby influencing cell growth and survival (26).

The PI3K-AKT pathway is pivotal in diverse cell types, influencing fundamental physiological processes like cellular metabolism, division, and differentiation (27–29). It is implicated in approximately one-third of human cancers, including CRC, impacting tumor cell growth, proliferation, programmed cell death, and metastasis (30, 31). The PI3K-AKT pathway serves as a signaling hub regulating critical cellular functions. Its dysregulation is a hallmark in cancer, including CRC, where aberrant activation influences key processes.

Human GI tumors prominently exhibit overexpression of PI3K/AKT proteins, correlating with tumor development and the emergence of immune tolerance (32, 33). and contributing to oncogenic signaling. Mutations in the PIK3CA gene, encoding the p110α catalytic subunit of PI3K, are notably observed in CRC (32). The presence of mutations in the PIK3CA gene accentuates the pathway’s role in CRC development.

Robust evidence from diverse experimental models underscores the significance of PI3K/AKT modulation in colon cancer. *In vivo* and *in vitro* studies consistently validate the pivotal role of PI3K/AKT activity in maintaining immune tolerance in colon cancer models and human CRC cell lines (34). The intricate web of interactions involving GSK-3β and β-catenin further highlights the nuanced regulatory mechanisms influencing immune tolerance. The activation of the PI3K/AKT/GSK-3β/β-catenin signaling cascade in CRC is implicated in fostering immune tolerance (35, 36). This knowledge provides a foundation for developing targeted interventions that disrupt these pathways, potentially enhancing anti-tumor immune responses.

The involvement of the PI3K-AKT pathway in liver metastasis of CRC mouse models extends beyond the local tumor environment. This intricate signaling cascade not only fuels primary tumor growth but also orchestrates a microenvironment conducive to immune evasion in distant metastatic sites. Through its ability to induce M2 macrophage polarization, the PI3K-AKT pathway creates an immunosuppressive milieu within the liver, fostering an environment conducive to the survival and growth of metastatic CRC cells. Moreover, the pathway’s influence on interleukin-10 (IL-10) levels is noteworthy. Elevated IL-10, orchestrated by PI3K-AKT activation, adds an additional layer to immune tolerance (37). IL-10, known for its anti-inflammatory properties, contributes to the suppression of immune responses, further facilitating the establishment of metastatic colonies in the liver.

Targeting PI3K/AKT has emerged as a viable strategy. Inhibiting the PI3K-AKT pathway has demonstrated effectiveness in diminishing the growth of GI tumors and countering immune tolerance. Emerging inhibitors, such as W922, a novel inhibitor, show promise in hindering the growth, migration, and invasion of CRC cells (38). This inhibition extends beyond mere tumor control, involving modulation of immune responses, providing a comprehensive therapeutic approach. Expanding the repertoire of potential treatments, PG2 from Astragalus membranaceus emerges as an intriguing candidate. Operating through the PI3K/Akt/mTOR/p70S6K signaling cascade, PG2 not only counteracts immune tolerance but also fosters immune activation (39). This dual action presents a holistic therapeutic approach, inhibiting tumor growth and metastasis while bolstering the host immune response against CRC (40, 41).

### 3.2 JAKs-STAT3 signaling pathway

The JAKs/STAT3 pathway plays a pivotal role in oncogenic processes, governing cell proliferation, differentiation, invasion, metastasis, and inflammation associated with cancer (42–44). A critical function involves signal transmission from cytokines (e.g., IL-6) and growth factors (e.g., TGF-α) to the cell nucleus. This activation commences upon the binding of cytokine ligands or growth factors to cell surface receptors, leading to the phosphorylation and dimerization of STAT3 by JAKs (JAK1, JAK2, JAK3, TYK2). Subsequently, facilitated translocation of STAT3 into the nucleus enables gene transcription (44). The hyperactivation of JAKs/STAT3 is closely linked to cancer immune tolerance (45), with elevated levels of phosphorylated STAT3 observed across various cancers, including breast (46, 47), lung (48), liver (49), GI (44, 50), prostate (51), fibrosarcoma (52), and melanoma (53). Phosphorylation of STAT3 is associated with increased PD-L1 expression on Treg cells (49, 54) and Indoleamine 2,3-dioxygenase 1(IDO1) on myeloid-derived suppressor cells (55), frequently observed in dedifferentiated cancer cells and infiltrating lymphocytes (45, 56).

The interaction between CXCL8 and STAT3 signaling holds significance, as CXCL8 enhances the infiltration of PD-L1(+) M2 macrophages while reducing the recruitment of PD-1(+) CD8(+) T cells in murine CRC models (57). This process is heavily reliant on the activation of STAT3 signaling. In human colon cancer cells, IL-22 emerges as a key regulator, upregulating PD-L1 expression through the activation of STAT3 signaling (58). This underscores the diverse array of cytokines capable of influencing immune responses and checkpoint molecule expression within the tumor microenvironment. Beyond cytokines and growth factors, additional activators fuel JAKs/STAT3 signaling. Increased GM-CSF triggers JAKs/STAT3 activation, inducing neutrophils to express B7-H4 in gastric cancer (50, 59). IL-6, a central mediator in immune and inflammatory responses, contributes to JAKs/STAT3 signaling, impeding dendritic cell maturation in colon carcinoma (60). Receptor tyrosine kinases, exemplified by the EGF receptor, serve as upstream activators, transmitting growth factor signals through JAKs/STAT3 activation. Moreover, FGFR2 amplifies PD-L1 expression via JAK/STAT3 signaling, culminating in apoptosis (61).

Strategies to inhibit the JAKs/STAT3 pathway prove instrumental in mitigating immune tolerance in cancer cells, with tangible outcomes observed in restraining tumor invasion and growth in xenografts (52, 54, 62). The confirmation of the pathway’s crucial role in cancer immune tolerance underscores its potential as a therapeutic target, offering prospects for novel interventions in the complex landscape of cancer biology.

### 3.3 NF-κB signaling pathway

NF-κB, a pivotal transcription factor, orchestrates immune responses (63). Beyond its involvement in lymphoid organ development, it modulates both innate and adaptive immunity. Moreover, its regulatory influence spans cytokine production, inflammation, and immune cell differentiation. The NF-κB family exhibits structural diversity, forming homo-/hetero-dimers such as p50/50, p52/p52, p50/RelA, and p52/RelA (64). Maintained in an inactive state by IκB in the cytoplasm, NF-κB undergoes dynamic structural changes upon activation. Cytokines, growth factors, or stress trigger NF-κB activation via IκB phosphorylation by the IKK complex. Subsequent ubiquitination and degradation of IκB liberate NF-κB, allowing its translocation into the nucleus, initiating gene transcription (65, 66).

NF-κB’s intricate involvement in cancer extends to various malignancies, including Glioblastoma (67), primary mediastinal large B-Cell lymphoma (68), and hepatocellular carcinoma (69). Genome profiling of nasopharyngeal carcinoma unveils a unique synergy between viral infection and NF-κB activation, contributing to immune escape (70). This synergy highlights NF-κB’s role in shaping the tumor microenvironment, influencing immune responses, and fostering conditions conducive to tumor progression. In gastric cancer, tumor-produced TNF-α activates NF-κB, inducing PD-L1 expression in mast cells (71). This fosters immune tolerance, exemplifying the diverse roles NF-κB plays in tumorigenesis. Cells within the tumor microenvironment (TME), especially immune cells, may induce NF-kB. Specifically, immune cells such as macrophages and T cells can release a variety of cytokines and chemokines in response to tumor antigens. These signaling molecules can activate the NF-kB pathway in tumor cells, thereby enhancing tumor cell survival, proliferation, and anti-apoptotic capabilities, supporting tumor growth and metastasis (72). Small molecules and biological agents that selectively inhibit NF-κB activation or disrupt its interactions with key signaling partners are under investigation. These interventions aim to halt aberrant NF-κB-driven processes, offering a nuanced approach to cancer treatment.

### 3.4 TGF-β/Smad signaling pathway

The TGF-β/Smad pathway, which is pivotal in GI cancer, involves TGF-βR I and TGF-βR II receptors (73). Located on the cell membrane, their interaction triggers the cascade. Upon TGF-β ligands binding, pathway activation initiates, showcasing its intricate cell signaling mechanism. Initially inhibitory to tumors, it transitions to a promoter of tumor progression and metastasis in advanced stages, underlining its dual nature (74, 75). Elevated TGF-β1 levels are evident in specific metastatic GI cancers. Functional correlation surfaces as inhibited TGF-β1 leads to reduced cell activity. This underscores TGF-β’s pivotal role in fostering metastatic characteristics, revealing its potential as a target for intervention in advanced cancer scenarios (76, 77).

TGF-β’s impact on immune tolerance is multifaceted. It suppresses Treg cells and various immune cell subsets, correlating positively with elements like IDO (78), hypoxia-inducible factor-1α (79) and Integrin αvβ6 (61). In gastric cancer, a positive correlation with FoxP3 is observed. Immunomodulation induced by TGF-β includes upregulation of PD-1, PD-L1 expression (80), vascular anomalies, and CD8(+) T cell suppression (63), underscoring its involvement in immune evasion. Furthermore, the upregulation of TGF-β plays a pivotal role in immune tolerance during GI metastasis. Its promotion of PD-1 and PD-L1 expression contributes to vascular abnormalities, forming a microenvironment that suppresses CD8(+) T cell-mediated anti-tumor responses (81). This signifies the intricate involvement of the TGF-β/Smad pathway in shaping the immunosuppressive landscape within metastatic GI cancers (82). Moreover, in pancreatic cancer liver metastasis, the TGF-β/SMAD signaling pathway not only induces EMT but also regulates the number of CSCs, enhancing stem cell properties to accelerate liver metastasis (83).

NCG, a nanodrug, encapsulates the TGF-β receptor inhibitor galunisertib. Exhibiting efficacy in colon cancer and liver metastasis treatment, it acts on multiple fronts. NCG inhibits myeloid-derived suppressor cell differentiation, induces M1-like polarization in tumor-associated macrophages, and disrupts the immunosuppressive barrier created by tumor-associated fibroblasts. The nanodrug’s impact extends to enhancing effector T cell infiltration, countering immunosuppression, and synergizing with anti-PD-L1 antibodies, presenting a promising avenue in GI cancer therapy (84). The TGF-β/Smad pathway, a linchpin in GI cancer, orchestrates a complex interplay influencing tumor progression, metastasis, and immune tolerance mechanisms. By dismantling immunosuppressive barriers and enhancing the efficacy of existing anti-cancer treatments, they pave the way for more effective strategies in the battle against GI cancers. The multifaceted role of the TGF-β/Smad pathway underscores its significance as a target for therapeutic intervention in the evolving landscape of GI cancer treatment.

### 3.5 Notch signaling pathway

The Notch family comprises four members, namely NOTCH1 to NOTCH4. This family is pivotal in orchestrating cell fate determination, influencing the developmental trajectory of cells (85). Each Notch protein possesses extracellular and transmembrane subunits. The interaction with ligands, such as Jagged-1 and DLL-4, triggers a two-step proteolytic cleavage process. This event leads to the liberation of the Notch intracellular domain (NICD), a crucial element in subsequent signaling cascades. Following cleavage, NICD relocates to the nucleus, where it exerts its influence. By binding with specific transcription factors, NICD modulates the transcription of Notch-responsive genes, thus regulating key cellular processes (44).

Mutations in Notch signaling significantly contribute to GI cancer progression. Notch signaling mutations play a pivotal role in orchestrating immune cell chemotaxis within the tumor’s immune microenvironment. This dynamic interaction influences the tumor’s ability to evade immune responses and promotes its progression. Elevated CCR9 expression is intricately linked to augmented NOTCH signaling in CRC. This heightened expression of CCR9 is associated with the establishment of central immune tolerance, impacting CRC metastasis, and chemoresistance (86). In xenograft mouse models, the correlation between CCR9 and NOTCH signaling provides insights into potential therapeutic interventions targeting this axis.

The interplay between Notch signaling and the tumor microenvironment plays a crucial role in tumor progression and therapy resistance. Notch signaling’s regulatory influence extends across various facets of tumor biology, including cell proliferation, angiogenesis, and metastasis (87). Consequently, targeting the Notch signaling pathway emerges as a promising avenue for novel cancer treatments. This approach addresses challenges like immune evasion and drug resistance in GI cancers, offering potential breakthroughs in cancer therapeutics.

### 3.6 PD-1/PD-L1 signaling pathway

The PD-1/PD-L1 system is pivotal in maintaining immune balance. Furthermore, PD-L1, prevalent in inactive lymphocytes and immune-privileged tissues, engages with PD-1, preventing hyperactive immune responses (88). During infections or inflammation, PD-L1 expression intensifies in hematopoietic, endothelial, and epithelial cells. Dendritic cells expressing PD-L1 in peripheral areas play a crucial role in fostering immune tolerance. In the thymus, PD-L1 facilitates the elimination of autoreactive naïve T-cells, ensuring self-tolerance. In peripheral areas, dendritic cells expressing PD-L1 contribute significantly to immune tolerance mechanisms (89). Moreover, in various cancers, malignant cells strategically exploit PD-1/PD-L1 interaction to evade immune surveillance. The expression of PD-L1 on cancer cells engages with PD-1 on tumor-infiltrating lymphocytes, inducing T-cell exhaustion (90). This phenomenon suppresses the anti-tumor immune response, fostering an immune-tolerant microenvironment that supports cancer progression.

PD-1/PD-L1 expression is closely associated with immunotolerance and poor prognosis in GI cancer (80, 91–93). Numerous studies have established a correlation between elevated PD-1/PD-L1 levels and adverse outcomes in patients with GI cancers. This association underscores the significance of the PD-1/PD-L1 pathway as a potential prognostic marker. Strategically targeting PD-1 or PD-L1 disrupts immune tolerance towards GI cancer cells. Clinical trials have demonstrated success with PD-1/PD-L1 inhibitors in GI cancers. Notably, a PD-1 inhibitor for CRC (94) and a PD-L1 inhibitor combined with C5aR1 in gastric cancer (95) have shown promising results, emphasizing the therapeutic potential of these interventions. Moreover, clinical trials provide compelling evidence of improved survival rates and reduced tumor progression in GI cancer patients treated with PD-1 inhibitors. Blocking PD-1 or PD-L1 has evolved into a pivotal therapeutic approach for GI cancers, highlighting its transformative impact on the clinical landscape. Combinatorial approaches, including the use of immune checkpoint inhibitors with traditional treatments, are being investigated to overcome resistance mechanisms and broaden the scope of therapeutic benefits. The success of blocking PD-1 or PD-L1 has positioned these inhibitors as crucial therapeutic tools in the oncological landscape. In summary, the shift toward immunotherapy in GI cancer treatment heralds a new era, demonstrating the efficacy of targeting the PD-1/PD-L1 axis. It marks a transformative breakthrough, reinvigorating the immune system’s ability to identify and eliminate cancer cells.

### 3.7 Wnt-β-catenin-IL-10 signaling axis

The Wnt/β-catenin signaling pathway is evolutionarily conserved, overseeing vital cellular processes such as proliferation, differentiation, migration, genetic integrity, apoptosis, and stem cell renewal. It serves as a molecular switch, tightly controlling gene expression and influencing cell fate decisions (96). This pathway employs canonical and noncanonical routes, with the fate determined by the stability of β-catenin within the cadherin protein complex. The canonical pathway involves β-catenin translocating into the nucleus, modulating gene expression, while the noncanonical pathways act independently of β-catenin, impacting cell polarity and movement. Within gut development and homeostasis, the Wnt signaling pathway assumes a central role (97). It orchestrates processes crucial for maintaining normal physiological conditions in the GI tract. Wnt signaling is pivotal in ensuring proper tissue organization, epithelial renewal, and the regulation of stem cells, contributing to the overall health and functionality of the gut (98).

Considering the importance of claudins in gastric cancer, such as the development of vaccines using CLDN6-mRNA, it is essential to highlight the relationship between Wnt signaling and the loss of epithelial cell differentiation. The Wnt signaling pathway plays a critical role in regulating cell proliferation, migration, and differentiation. Aberrations in this pathway can lead to disrupted epithelial cell differentiation, a hallmark of cancer progression. Claudins, particularly CLDN6, are integral to maintaining cell-cell adhesion and epithelial integrity (99). Dysregulation of Wnt signaling can compromise the expression and function of claudins, thereby facilitating the loss of epithelial characteristics and promoting a more invasive and metastatic tumor phenotype (100). Understanding this connection underscores the potential of targeting Wnt signaling and claudin expression as therapeutic strategies in gastric cancer treatment.

Wnt signaling aberrations are strongly associated with cancers, particularly colon cancer. Genome-wide studies reveal significant anomalies in Wnt signaling among colon cancer patients, suggesting its pivotal role in the initiation and progression of the disease (101, 102). Dysregulation in this pathway contributes to uncontrolled cell growth and tumor formation. CRC is intricately linked to the loss of immune tolerance to gut flora, intensified by chronic intestinal inflammation. The Wnt-β-catenin-IL-10 signaling axis in intestinal antigen-presenting cells responds to symbiotic flora, influencing levels of the suppressive cytokine IL-10 and pro-tumor inflammatory factors. Initiating the Wnt signaling pathway strategically emerges as a potential biological intervention to mitigate inflammation and slow colon cancer progression. Recent studies demonstrating protective effects in mice through the Wnt-β-catenin-IL-10 axis highlight its therapeutic potential. This approach may offer a targeted strategy to modulate immune responses and inhibit the development of CRC (103). Exploring the therapeutic implications of Wnt signaling in CRC opens avenues for targeted interventions. Understanding the intricate network of Wnt-β-catenin interactions and their impact on immune responses allows for the development of novel therapeutic strategies. Future research may focus on refining techniques for modulating Wnt signaling specifically within the tumor microenvironment, paving the way for more effective and tailored treatments.

## 4 Molecules associated with immune tolerance in metastatic GI cancers

### 4.1 Indoleamine 2,3-dioxygenase

IDO1, an immunomodulatory enzyme, plays a pivotal role in CRC. It suppresses T cell activation and weakens regulatory T cell (Treg) function, fostering immune tolerance within the tumor microenvironment (104, 105). IDO1’s involvement in cancer development is multifaceted. Operating through the Kyn pathway (105), it activates β-catenin, TGF-β, and PI3K-Akt signaling pathways (34, 78, 106). This activation amplifies cancer cell proliferation, impedes apoptosis, and significantly contributes to colon cancer progression. Notably, elevated IDO1 expression at the tumor invasion front correlates with disease progression, serving as an independent prognostic marker for CRC (107).

Furthermore, the IDO-Kynurenine-AhR axis emerges as a key orchestrator of immune tolerance. Elevated serum Kyn-to-Tryptophan ratio, indicative of IDO activation, holds promise as a potential CRC screening marker. Kyn, an AhR agonist, facilitates Treg cell differentiation and upregulates PD-1 expression in CD8+ T cells (34, 78, 106). Inhibiting this axis demonstrates therapeutic potential, mitigating immune tolerance and suppressing colitis-associated colon cancer. Moreover, the intricate PrP(C)-ILK-IDO1 axis also emerges as a noteworthy player in CRC. PrP(C), highly expressed in CMS4 tumors, intricately regulates CMS4-specific genes. As a proximal effector of PrP(C), Integrin Linked Kinase (ILK) plays a pivotal role, influencing the expression and activity of IDO1 (108). Besides, IDO1’s *in vitro* expression in CRC is contingent on IFN-γ stimulation. The interplay between IFN-γ and IDO1 highlights the dynamic regulation of immune responses in the context of CRC (107).

Considering the inhibitory potential of IDO, blocking its activity stands out as a promising strategy to impede tumor growth. Compounds like 1-methyltryptophan (1-MT) and (-)-epigallocatechin gallate (EGCG) exhibit efficacy in reducing aberrant crypt foci and β-catenin-accumulated crypts, both commonly associated with IDO protein overexpression (109). These compounds showcase therapeutic potential by disrupting the pro-tumorigenic effects mediated by IDO. The chemopreventive potential of IDO inhibitors, exemplified by 1-MT, and natural compounds like EGCG, holds promise in suppressing preneoplastic lesions in the colon. These compounds, with their ability to modulate IDO activity, represent innovative strategies for colon cancer chemoprevention (110). Therefore, exploring their efficacy in clinical settings may provide valuable insights into preventive measures against the early stages of colon carcinogenesis. The multifaceted role of IDO1 in CRC, from immune tolerance and prognostic implications to intricate molecular pathways and potential therapeutic interventions, underscores the complexity of this enzyme in the context of cancer biology.

### 4.2 Human leukocyte antigen G and E

HLA-G, a non-classical HLA class I antigen, is predominantly expressed in extravillous cytotrophoblasts during normal pregnancy. In normal conditions, its expression is confined to immune-privileged tissues (111). In various cancer types, there is significant aberrant induction of HLA-G expression. This abnormal expression is strongly correlates with tumor metastasis and poor prognosis (112, 113). HLA-G functions as both an immune tolerance and tumor-promoting factor (114). Extensive studies have established HLA-G as a distinct immune checkpoint. Its immunotolerance functions include suppressing LT CD8+ and NK cells’ cytolytic effects, inhibiting LT CD4+ and dendritic cells’ maturation (20), and enhancing Th2 cytokines (113). In CRC, undifferentiated cells release HLA-G at the invasion front. This induces a transformation in macrophages towards the M2 phenotype (SPP1+ macrophages). SPP1+ macrophages attract regulatory T (Treg) and CD8+ T cells into CRC tissues, leading to anti-tumor immune resistance and depletion of CD8+ T cells (115, 116).

HLA-E, a nonclassical HLA class I molecule, contributes to immune escape in GC cell lines (117). Various immune cells, including malignant tumor cells, specific endothelial subcluster, mucosal-associated invariant T cells, T cell-like B cells, plasmacytoid dendritic cells, macrophages, monocytes, and neutrophils, are involved in HLA-E interactions (118). Patients with advanced-stage GC exhibit elevated soluble HLA-E levels, correlating with shorter overall survival (OS) compared to those with lower levels (117). Current research emphasizes the significant clinical impact of soluble HLA-E in immune escape of GC cells. Patients with elevated soluble HLA-E levels in advanced-stage GC tend to experience shorter OS compared to those with lower levels. Soluble HLA-E is identified as a potential clinical marker in GC patients. In summary, research emphasizes the significant clinical impact of soluble HLA-E in immune escape of GC cells. Patients with elevated soluble HLA-E levels in advanced-stage GC tend to experience shorter OS compared to those with lower levels. Soluble HLA-E is identified as a potential clinical marker in GC patients.

These findings collectively underscore the multifaceted role of non-classical HLA antigens, particularly HLA-G and HLA-E, in cancer progression and immune escape mechanisms. Further exploration of these pathways may offer valuable insights for developing targeted therapeutic strategies in cancer treatment.

### 4.3 Glycoprotein A repetitions predominant

GARP, encoded by the Lrrc32 gene, acts as a transmembrane receptor for latent TGF-β. The GARP complex consists of a TGF-β dimer and latency-associated peptides (119). This interaction plays a pivotal role in regulating TGF-β signaling pathways. GARP collaborates with integrin to facilitate the release of active TGF-β from the cell surface (120). This interaction enhances the suppressive capabilities of Treg cells, contributing significantly to immune regulation. The interplay between GARP and integrin sheds light on the dynamic mechanisms governing TGF-β release. Various environmental factors, including heat, acidic conditions, and interactions with integrins, act as triggers for the release of mature, biologically active TGF-β from the GARP complex (121).

Notably, GARP is expressed on activated Treg cells, serving as a distinctive marker (122). Its primary function involves binding to latent TGF-β, suggesting a crucial role in modulating Treg cell activities (123, 124). Elevated GARP expression is correlated with increased populations of FOXP3+ Treg and CD4+ T cells in diverse cancers such as gastric, colon, lung, and breast cancers (125–127). Importantly, heightened GARP levels are linked to poorer patient outcomes in these malignancies. Exploring the relationship between GARP expression and cancer progression provides insights into potential therapeutic interventions. Within the GI tract, GARP plays a critical role in maintaining systemic immune balance (127). Its essential contribution to the transformation of naïve CD4+ T cells into Treg cells highlights its significance in shaping immune responses in this specific context. In the realm of CRC therapy, GARP emerges as a potential therapeutic target. Its absence in Treg cells is associated with improved antitumor immunity (128), underscoring its importance in both Treg function and tissue-specific immune responses.

GARP’s crucial role in the GI tract extends to its involvement in cancer therapy, particularly in CRC. As a potential therapeutic target, inhibiting GARP expression in Treg cells holds promise for promoting improved antitumor immunity. The absence of GARP in Treg cells is associated with a shift towards a more immune-responsive environment, emphasizing its intricate involvement in orchestrating tissue-specific immune responses (120). In conclusion, GARP’s multifaceted functions, from being a key component of the TGF-β superfamily to influencing Treg cell activities and immune responses in cancer contexts, position it as a pivotal player in both physiological and pathological conditions. Further research into the intricate mechanisms governed by GARP is essential for unlocking its full therapeutic potential in immune modulation and cancer treatment.

### 4.4 Common lymph endothelial and vascular endothelial receptor-1

Within the intricate landscape of the tumor microenvironment, macrophages emerge as pivotal orchestrators. In this complex milieu, their dynamic interactions, often with lymphatic vessels, wield significant influence over diverse cancer outcomes. As sentinels of the immune system, these cells navigate the complex terrain of tumors, shaping the milieu in ways that can either impede or foster cancer progression. GI cancers face the formidable impact of Tumor-Related Macrophages (TAMs). These macrophages, endowed with remarkable versatility, can adopt M1 or M2 phenotypes, or a blend of both, exerting nuanced effects on the tumor microenvironment. The versatility lies in their ability to adopt M1 or M2 phenotypes, or a blend of both, exerting nuanced effects on the tumor microenvironment (129, 130). Their presence and functional plasticity underscore the intricate interplay between the immune system and the progression of GI cancers.

At the crossroads of immune regulation and vascular dynamics lies Clever-1, a molecule intricately woven into cancer scenarios. Situated on immunosuppressive M2 macrophages and specific endothelial cells, Clever-1 manifests in critical locales such as lymphatic vessels, liver, spleen sinusoids, and high endothelial venules (HEVs) (131, 132). Its multifaceted involvement in cellular processes, ranging from cell movement to tissue remodeling, paints Clever-1 as a key player in the complex orchestration of cancer progression (132, 133). The strategic placement of Clever-1 on immunosuppressive M2 macrophages and specific endothelial cells positions it strategically in critical locales such as lymphatic vessels, liver, spleen sinusoids, and HEVs. The absence of Clever-1 gene expression emerges as a formidable barrier to solid tumor growth. In a fascinating interplay with the body’s own defense mechanisms, this lack stimulates the activation of CD8+ antitumor cells. This effect echoes the outcomes observed with PD-1 checkpoint inhibitors, hinting at Clever-1’s pivotal role as a regulator of the delicate balance between tumor proliferation and immune surveillance.

In the quest for more effective cancer therapies, a synergistic treatment approach beckons. To this end, combining anti-Clever-1 and anti-PD-1 treatments emerges as a promising strategy with potential synergistic benefits (134). This dual targeting of these pathways holds the prospect of enhanced efficacy, particularly in combating aggressive and treatment-resistant tumors. This strategic alliance may unlock new avenues for therapeutic interventions, elevating the prospects for improved patient outcomes. Moreover, the expression levels of Clever-1 become a critical determinant in the landscape of immunotherapy response. High Clever-1 expression emerges as a foreboding sign, associated with poorer responses to immunotherapy (92). Insights from The Cancer Genome Atlas’s pan-cancer study draw a compelling correlation between elevated Clever-1 expression and shortened survival in cancer patients (133). Unraveling the intricacies of this association holds the potential to refine immunotherapeutic strategies and improve prognostic precision in the realm of cancer treatment.

### 4.5 Interferon regulatory factor 8/Osteopontin

T cell activation is a complex process involving the transformation of naive CD8+ T cells into effector cytotoxic T cells. This transition is characterized by significant changes in gene expression, orchestrated by T cell-specific transcription factors like T-bet, Eomes, and IRF8 (135). These transcription factors play a pivotal role in marking the molecular shift, ultimately influencing the functionality of activated T cells (136). Initially recognized for its involvement in myeloid cell differentiation, IRF8 emerges as a crucial player in the activation and differentiation of CD8+ T cells. Beyond its myeloid role, IRF8 acts as an antagonist of OPN, inhibiting OPN expression in colon epithelial cells. The correlation between decreased IRF8 and increased OPN expression in colon carcinoma underlines the significance of IRF8 in immune checkpoint mechanisms within GI cancers (137, 138).

OPN, a sialic acid-rich phosphoprotein within the extracellular matrix, interacts with integrin and CD44 receptors (139, 140). CD44, featuring extended isoforms, binds with hyaluronate, influencing tissue structure, cell aggregation, and movement (139, 141). OPN’s role extends to modulating cell proliferation, metastasis, and apoptosis signaling. Notably, decreased OPN expression is linked to diminished proliferation and metastasis in GI cancers, highlighting its critical regulatory role (142–145). In the clinical context of GI cancers, elevated OPN levels serve as significant indicators of advanced tumor stages and poorer prognoses. Beyond its prognostic value, OPN emerges as an additional immune checkpoint in GI cancers. Its ability to contribute to tumor immune tolerance and evasion underscores the pivotal role OPN plays in shaping the immunological landscape within the GI tract, influencing disease progression and treatment outcomes.

The synergistic interplay between IRF8, OPN, and T cell activation intricately contributes to the mechanisms of immune evasion in GI cancers. The dysregulation, characterized by decreased IRF8 and increased OPN expression in colon carcinoma cases, exemplifies the orchestrated orchestration of these molecules in shaping tumor immune tolerance and evasion (145). Both myeloid and tumor cells strategically exploit the suppression of IRF8 expression to enhance OPN levels. This manipulation acts as an immune checkpoint mechanism, impeding the activation of cytotoxic T lymphocytes (CTLs) and fostering an environment conducive to immune evasion within the GI cancer microenvironment.

### 4.6 T-cell immunoglobulin domain and mucin domain-3 and carcinoembryonic antigen cell adhesion molecule 1

Initially discovered in CD4+ T cells producing interferon-gamma (IFN-γ), TIM-3 has emerged as a key player in regulating T-cell function (146). Notably, its expression is significantly correlated with T-cell dysfunction. When the interaction between TIM-3 and its ligand is blocked, there is a restoration of IFN-γ production, suggesting a potential therapeutic avenue for modulating T-cell responses. Within Treg cells, a specific subset expresses TIM-3, particularly in tumor-infiltrating lymphocytes. This unique expression pattern is closely associated with immunosuppressive capabilities. Importantly, the presence of TIM-3 in Treg cells is indicative of a poorer prognosis in cancer patients, particularly those with tumors (147).

As a member of the Carcinoembryonic antigen (CEA) family, CEACAM1 serves as a valuable biomarker, particularly on activated T cells. Widely recognized for its role in monitoring relapse and chemotherapy responses in CRC patients, CEACAM1 also acts as a regulator of apoptosis in colon cells, functioning as a tumor suppressor. Loss of CEACAM1 is a common occurrence in the early stages of CRC tumorigenesis (148), indicating its potential role as a critical factor in disease initiation. Paradoxically, increased CEACAM1 expression in metastatic colon cancer suggests a dual role, implicating its involvement in CRC progression (149).

The interaction between TIM-3 and CEACAM1 unveils a sophisticated regulatory network that impacts both autoimmunity and anti-tumor immunity. Activation of TIM-3 contributes to immune tolerance and instigates T cell exhaustion, while CEACAM1 acts as a heterophilic ligand for TIM-3, playing a crucial role in inducing T-cell exhaustion. This intricate interplay between TIM-3 and CEACAM1 holds significant implications for modulating immune responses in the context of cancer and autoimmune disorders. High expression levels of TIM-3 and CEACAM1 in circulating CD8+ T cells serve as valuable indicators of CRC progression. Notably, the TIM-3+CEACAM1+ CD8+ T cell subset emerges as the most dysfunctional group with minimal IFN-γ production. The simultaneous presence of these markers is associated with advanced cancer stages and is identified as an independent risk factor for CRC (150). Consequently, targeting both CEACAM1 and TIM-3 concurrently holds promise as a strategy to enhance anti-tumor immune responses in CRC, as evidenced by successful outcomes in CRC mouse models (151). This dual blockade presents a potential avenue for innovative immunotherapeutic interventions in CRC.

### 4.7 Caspases-1 and -12

The severity of H. pylori-induced gastritis exhibits a positive correlation with the risk of cancer development, particularly in the context of intestinal cancer (152). Persistent inflammation caused by H. pylori infection becomes a critical factor in initiating and promoting tumorigenesis within the GI tract (153). Inflammatory bowel diseases, including ulcerative colitis and Crohn’s disease, are closely associated with an elevated risk of CRC. The chronic inflammation characteristic of these diseases creates a conducive environment for the initiation and progression of CRC, highlighting the intricate link between inflammation and cancer.

Inflammatory caspases play a pivotal role in preserving intestinal equilibrium, and disruptions in the caspase pathway are identified as fundamental elements in inflammation-associated tumorigenesis. The delicate balance maintained by these caspases is crucial for preventing aberrant cell behavior and promoting tissue homeostasis within the GI system. Specifically, inflammatory caspases, with caspase-1 playing a notable role, regulate the production of proinflammatory cytokines such as IL-1b and IL-18 (154). These cytokines are pivotal factors in the development of gastritis and associated cancers. The dysregulation of this intricate cytokine network underscores the importance of understanding and targeting caspase-mediated pathways for therapeutic interventions. Within the inflammasome, caspase-1 activation is a critical event, and various inflammasomes (NLRP1, NLRP3, IPAF, NAIP5) depend on the adaptor protein ASC to trigger and enhance caspase-1 activity (155). This intricate molecular machinery highlights the complexity of inflammasome regulation, linking its activation to caspase-1 and the crucial role played by ASC in orchestrating these inflammatory responses. Functioning as an inflammasome repressor, caspase-12 acts as a ‘brake’ on caspase-1’s activity (156, 157). Regulating caspase-1 through caspase-12 is vital for maintaining immune tolerance in the gut (158). Disruption in this delicate balance can lead to severe inflammation, providing a mechanistic link between caspase-12 dysfunction and inflammation-induced GC or CRC (158, 159).

### 4.8 Cell division control protein 42

The dynamic reorganization of the actin cytoskeleton stands as a critical determinant influencing alterations in cellular morphology. This intricate process is particularly pivotal in the development of lamellipodia and filopodia, structures essential for the directional movement and invasive capabilities of tumor cells (160). In essence, the dynamic changes in cellular morphology are intricately linked to the reorganization of actin filaments, underscoring its profound impact on cellular behavior and function. Tumor metastasis hinges significantly on the stimulation of cytoskeletal proteins, serving as a crucial molecular event. This stimulation marks the initiation of either an invasive or metastatic phase in tumor progression. Moreover, the persistence of tumor cells post-extravasation is occasionally facilitated by these cytoskeletal alterations. The motile behavior of cancer cells is intricately governed by Rho GTPases, with Cdc42 emerging as a central player (161).

As a member of the Ras superfamily, Rho GTPases function as dual-state molecular switches, activated by GTP and deactivated by GDP (162). Their role in orchestrating cancer cell motility is indispensable, as they intricately regulate cytoskeletal dynamics, influencing cell migration and invasion. The intricate activity of Rho GTPases is subject to precise regulation, responding dynamically to various triggers. Growth factors, cytokines, and cell-cell or cell-extracellular matrix interactions play pivotal roles in modulating Rho GTPase activity. These molecular switches are recognized as key signaling conduits, participating in numerous pathways within eukaryotic cells.

Within the context of CRC, Cdc42, a prominent member of the Rho GTPase family, assumes a central role in disease progression (163, 164). Its influence extends beyond conventional pathways, impacting the activation of CD8+ T cells, facilitating immune evasion, and directly contributing to oncogenic activities (165, 166). Moreover, Cdc42’s significance transcends cancer cell biology, playing a crucial role in maintaining the equilibrium and stability of Treg cells. The homozygous deletion of Cdc42 in Treg cells leads to a reduction in their population, decreased stability, and the onset of early, lethal inflammatory diseases in CRC (167). Targeting Cdc42 holds promise in disrupting Treg cell stability and amplifying anti-tumor T-cell immunity (168). Additionally, the association between elevated circulating CDC42 levels and adverse clinical outcomes underscores its potential as a prognostic marker in CRC (169, 170), emphasizing its importance in both cancer biology and immune regulation.

## 5 Treatment strategies

In animal experiments focused on gastric cancer peritoneal metastasis, Tranilast has shown efficacy by inhibiting tumor growth and fibrosis within the tumor immune microenvironment (TME) (171). It achieves this by reducing the migration ability of M2 macrophages and the infiltration of mast cells, thus ameliorating the immunosuppressive microenvironment. Similarly, in the context of colitis-associated metastatic colorectal tumors, RQ-15986, a selective EP4 antagonist of prostaglandin E2, demonstrates therapeutic benefits. It alleviates inflammation, inhibits cell proliferation, and modulates the expression of indoleamine 2,3-dioxygenase, addressing key aspects of tumor progression (172).

Further into the realm of molecular interventions, TEX-miR-34a emerges with its dual role: delivering miRNA and possessing potential for triggering anti-tumor immune responses (173). Another noteworthy miRNA, miR-448, targets tumor suppression by enhancing CD8(+) T cell responses through the inhibition of IDO1 expression (174). This strategy outlines a promising avenue for combinational immunotherapy in treating metastatic GI cancers.

Moreover, exosomes play a critical role as communicators between tumor and host cells, highlighting the significance of the molecular dialogue in cancer dynamics. Among the molecular targets, heterogeneous nuclear ribonucleoprotein I (hnRNPI) stands out for its role in the development of aggressive colorectal cancer, suggesting its potential as a therapeutic target (175).

The text also underscores the efficacy of combination therapies, such as the use of C5aR1 blockers with PD-1 inhibitors (95), T7 - MB with 5-FU (176), and the combination of the NAMPT inhibitor APO866 with the IDO-specific inhibitor L-1-methyl-tryptophan (L-1MT) (109). These combinations are instrumental in preventing tumor metastasis and achieving synergistic tumor eradication.

In addition to these therapeutic strategies, several innovative treatments exhibit strong anti-tumor activity (177). These include the PDL1-targeting vaccine (178), the xenogeneic polyantigenic vaccine (XPV) (179), dendritic cells (DCs)-based vaccines (180), a genetic vaccine platform based on DNA electroporation and adenovirus (Ad) (181), and the telomerase reverse transcriptase vaccine (182). Collectively, these approaches showcase the evolving landscape of treatments with significant potential in combating metastatic gastrointestinal tumors.

## 6 Conclusion and perspectives

In concluding our review, it is imperative to acknowledge the limitations in the current body of research on immunotolerance in gastrointestinal cancers. Predominantly, studies have been confined to basic laboratory research, with a relative dearth in clinical application investigations. For instance, while multiple potential mechanisms of immune evasion have been identified in cellular and animal models, their validation and application in human patients remain significantly limited. Furthermore, the literature presents conflicting results; where some studies report positive effects of specific immune checkpoint inhibitors in treating gastrointestinal cancers, others do not observe significant therapeutic outcomes. These discrepancies could stem from variations in sample sizes, study designs, or patient demographics. Despite research efforts focusing on the mechanisms of immunotolerance in gastrointestinal cancers, substantial knowledge gaps persist. Critical questions remain unanswered, particularly regarding the differences in immunotolerance mechanisms across various types of gastrointestinal cancers, such as colorectal and gastric cancers, where comparative studies are notably scarce.

The ongoing battle against GI cancers, encompassing malignancies in the digestive tract and its accessory organs, is marked by both significant challenges and emerging opportunities. Despite notable progress in early detection and traditional treatment modalities, the persistent high mortality rate, particularly in late-stage inoperable GI cancers, underscores an urgent need for innovative therapeutic strategies. A critical insight into the biology of these cancers reveals that immune tolerance plays a pivotal role in their progression. This phenomenon, where the immune system paradoxically fails to recognize and combat tumor cells, enables cancer cells to masquerade as normal tissue, thereby exploiting the body’s natural tolerance mechanisms. The adaptation of cancer cells to mimic the body’s own cells is not just a survival tactic but a sophisticated strategy to proliferate unchallenged. This understanding has shifted the paradigm of cancer research and treatment, highlighting the need for interventions that directly target the mechanisms of immune evasion. In the complex interplay of the cancer immunity cycle, where immune cells are expected to recognize and attack malignancies, GI cancers disrupt this process through immune evasion, particularly during the equilibrium phase of cancer-immune interaction. The ability of cancer cells to induce a state of immune tolerance, akin to physiological tolerance in normal tissues, represents a significant hurdle but also a potential target for novel treatments. The current focus on immunotherapy is a testament to this new direction in cancer treatment, emphasizing the potential of the immune system as a powerful ally in this fight.

**Figure 1:**
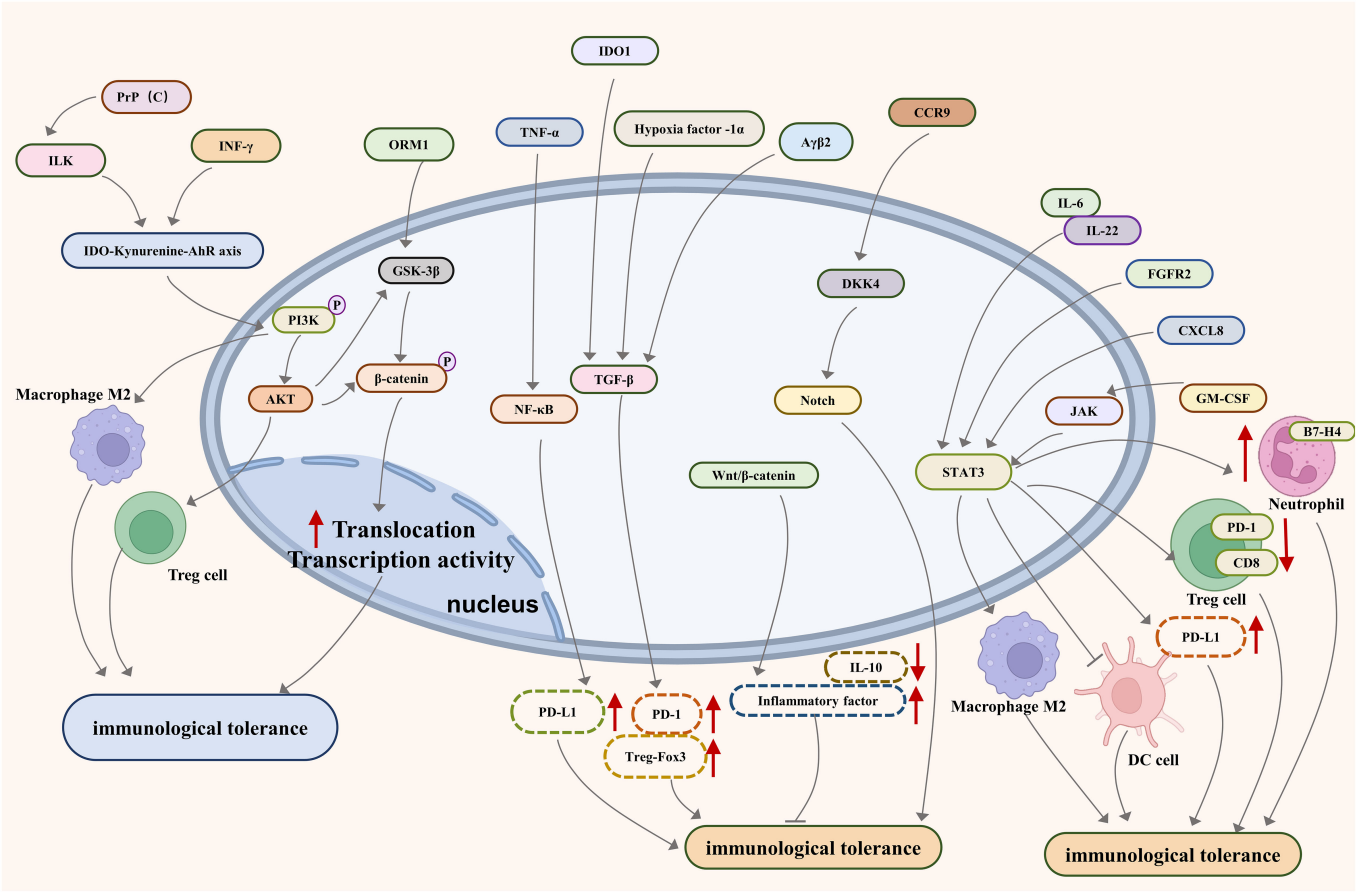
Major pathways associated with immune tolerance in metastatic gastrointestinal cancer.

**Figure 2:**
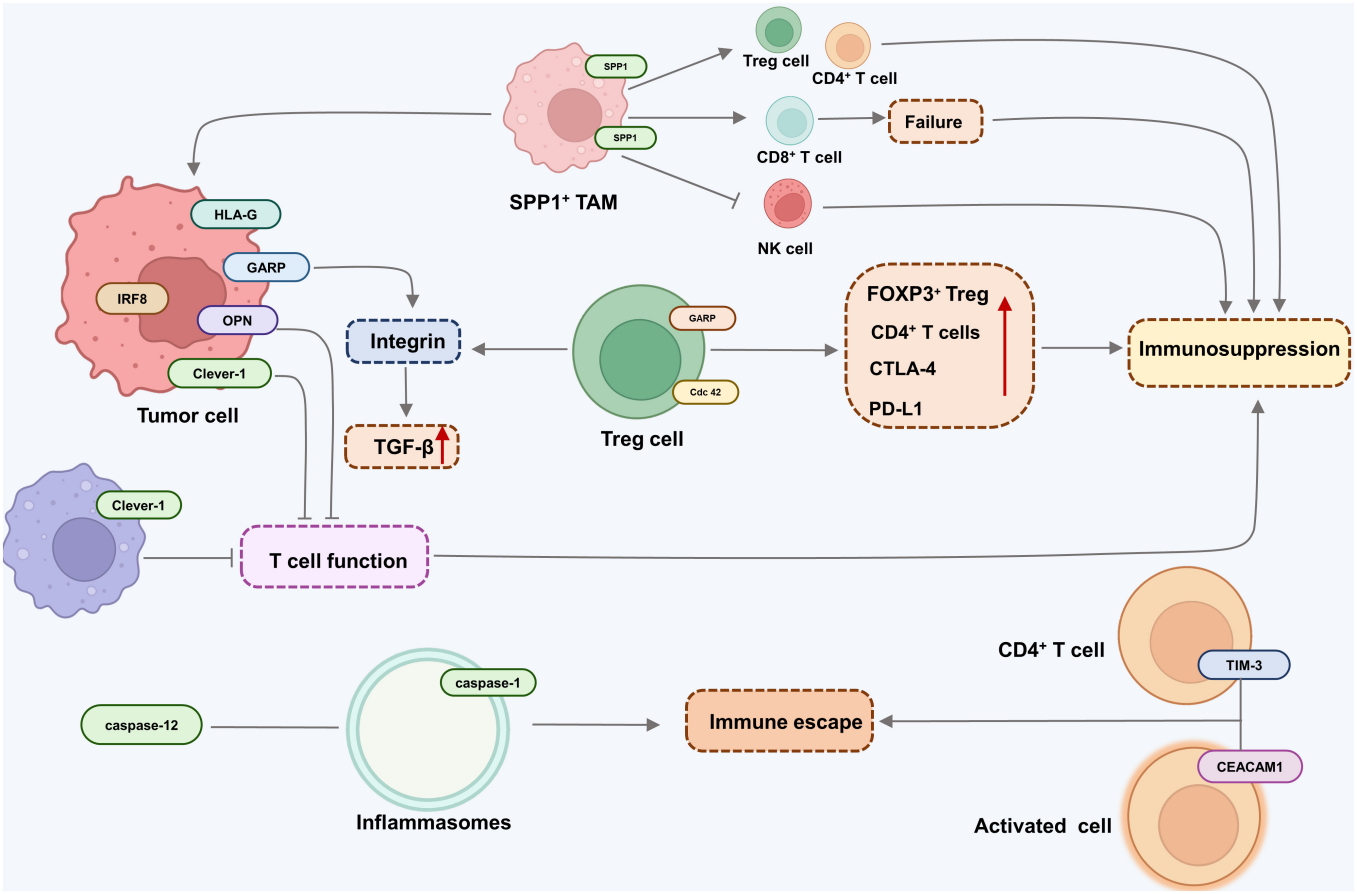
Major molecules associated with immune tolerance in metastatic gastrointestinal cancer.

Looking forward, the development of targeted immunotherapies presents a promising avenue. These therapies are designed to revitalize the immune system, enabling it to identify and destroy cancer cells effectively. The exploration of cellular signaling pathways such as PI3K/AKT, JAKs-STAT3, NF-kB, TGF-b/Smad, Notch, PD-1/PD-L1, and Wnt-bcatenin- IL-10 (**Figure 1**), alongside the study of relevant molecules like IDO, HLA-G/E, GARP, Clever-1, IRF8/OPN, TIM-3, CEACAM1, Cdc42, and Caspases-1 and -12 (**Figure 2**), opens new doors for understanding and disrupting the mechanisms of immune tolerance in GI cancers. Moreover, the integration of advanced genomic and proteomic technologies in cancer research offers an unprecedented opportunity to decode the molecular complexities of immune evasion. By harnessing big data and artificial intelligence, researchers can identify novel biomarkers and therapeutic targets, paving the way for personalized and more effective treatments. The prospect of combining immunotherapy with other treatment modalities, such as chemotherapy and radiation, also holds great potential. Such combination therapies could potentially overcome the limitations of single-modality treatments, offering a more holistic approach to cancer management.

In conclusion, the fight against GI cancers is entering a new era marked by a deeper understanding of cancer biology and the immune system’s intricacies. The future lies in leveraging this knowledge to develop innovative, targeted therapies that not only treat but also prevent the progression of these malignancies. The journey ahead is challenging but filled with potential, promising a new horizon in the battle against cancer.

## Author contributions

QG: Conceptualization, Funding acquisition, Investigation, Resources, Software, Visualization, Writing – original draft, Writing – review & editing. YL: Writing – review & editing, Conceptualization, Writing – original draft. YJL: Software, Writing – review & editing. HL: Visualization, Writing – original draft, Software. LL: Conceptualization, Writing – original draft. DC: Writing – original draft, Conceptualization, Visualization. CP: Supervision, Writing – original draft.
